# Systemic Tumor Suppression via Macrophage‐Driven Automated Homing of Metal‐Phenolic‐Gated Nanosponges for Metastatic Melanoma

**DOI:** 10.1002/advs.202207488

**Published:** 2023-04-18

**Authors:** Xue Liao, Guidong Gong, Mengyuan Dai, Zhenyu Xiang, Jiezhou Pan, Xianglian He, Jiaojiao Shang, Anna Maria Blocki, Zongmin Zhao, C. Wyatt Shields, Junling Guo

**Affiliations:** ^1^ BMI Center for Biomass Materials and Nanointerfaces College of Biomass Science and Engineering Sichuan University Chengdu Sichuan 610065 China; ^2^ National Engineering Laboratory for Clean Technology of Leather Manufacture Sichuan University Chengdu Sichuan 610065 China; ^3^ School of Biomedical Sciences Faculty of Medicine The Chinese University of Hong Kong Hong Kong SAR 999077 China; ^4^ Department of Pharmaceutical Sciences College of Pharmacy University of Illinois at Chicago Chicago IL 60612 USA; ^5^ Department of Chemical and Biological Engineering University of Colorado Boulder CO 80303 USA; ^6^ Bioproducts Institute Department of Chemical and Biological Engineering University of British Columbia Vancouver BC V6T 1Z4 Canada; ^7^ State Key Laboratory of Polymer Materials Engineering Sichuan University Chengdu Sichuan 610065 China

**Keywords:** acid‐induced drug release, macrophages, metal‐phenolic gatekeepers, nanosponges, tumor‐targeted therapy

## Abstract

Cell‐based therapies comprising the administration of living cells to patients for direct therapeutic activities have experienced remarkable success in the clinic, of which macrophages hold great potential for targeted drug delivery due to their inherent chemotactic mobility and homing ability to tumors with high efficiency. However, such targeted delivery of drugs through cellular systems remains a significant challenge due to the complexity of balancing high drug‐loading with high accumulations in solid tumors. Herein, a tumor‐targeting cellular drug delivery system (MAGN) by surface engineering of tumor‐homing macrophages (M*φ*s) with biologically responsive nanosponges is reported. The pores of the nanosponges are blocked with iron‐tannic acid complexes that serve as gatekeepers by holding encapsulated drugs until reaching the acidic tumor microenvironment. Molecular dynamics simulations and interfacial force studies are performed to provide mechanistic insights into the “ON‐OFF” gating effect of the polyphenol‐based supramolecular gatekeepers on the nanosponge channels. The cellular chemotaxis of the M*φ* carriers enabled efficient tumor‐targeted delivery of drugs and systemic suppression of tumor burden and lung metastases in vivo. The findings suggest that the MAGN platform offers a versatile strategy to efficiently load therapeutic drugs to treat advanced metastatic cancers with a high loading capacity of various therapeutic drugs.

## Graphical abstract

A tumor‐targeting cellular drug delivery system is developed by surface‐engineering macrophages with biologically responsive nanosponges gated by polyphenol‐based supramolecular “gatekeepers”. The nanosponges enables a high‐loading content of drugs, robust attachment to cellular surfaces, and drug‐controlled release in an acidic tumor microenvironment, which results in the efficient tumor‐targeting delivery of chemotherapeutic drugs and the systemic suppression of tumors in vivo.

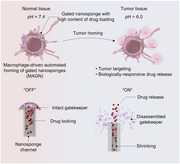

## 1 Introduction

Cell‐based drug delivery systems have been actively explored due to their advantages of tissue‐specific tropism, long circulation, and high biocompatibility.^[^ ^1^, ^2^, ^3^, ^4^, ^5^, ^6^ ^]^ Due to their ability to infiltrate inflamed tissues with remarkable specificity, surpassing that of free nanoparticles,^[^ ^7^ ^]^ cellular delivery systems have gained notable attention for delivering a range of drugs to solid tumors. Still, the targeted delivery of drugs with living cells remains a challenge due to the needs of maintaining cellular viability,^[^ ^8^ ^]^ achieving high loading without compromising chemotaxis,^[^ ^9^ ^]^ and avoiding uncontrolled drug diffusion,^[^ ^10^, ^11^ ^]^ each of which generally requires highly complex and time‐consuming preparation processes to remedy. One approach to achieving high drug loading is the encapsulation of drugs into the inner volume of cells by diffusion,^[^ ^12^ ^]^ endocytosis,^[^ ^13^, ^14^, ^15^ ^]^ or electroporation.^[^ ^16^, ^17^, ^18^ ^]^ However, these strategies usually enhance drug loading, which can result in cell membrane alterations or interfere with cell functionalities, subsequently reducing chemotactic migration to solid tumors.^[^ ^1^, ^19^ ^]^

Attachment of therapeutic molecules to the cellular surfaces has been leveraged for cell‐based drug delivery (referred to as “backpacking” or “hitchhiking”).^[^ ^20^, ^21^, ^22^, ^23^ ^]^ A range of payloads including proteins, therapeutic compounds, and nanoparticles have been attached to cellular surfaces for various therapeutic applications.^[^ ^24^, ^25^, ^26^, ^27^ ^]^ Particularly, particle‐based hitchhiking systems were mostly designed to encapsulate payloads in synthetic nanoparticles, with the content of payloads integrated on the cellular carrier being significantly low due to the complex fabrication of hitchhiked nanoparticles and their limited loading capacity. An interesting exception was demonstrated by Mitragotri and co‐workers, where the authors used polyphenol‐mediated interactions to integrate various biomolecules on the surfaces of cells (referred to as “Cellnex”),^[^ ^28^ ^]^ offering a versatile platform for a wide range of cell‐based therapies. However, the direct interaction between polyphenols and biomolecules generally leads to their reduced bioactivity. Additionally, the specific functionalization ratio of polyphenols on various biomolecules needs to be optimized on a case‐by‐case basis owing to the diverse molecular compositions and properties of the encapsulated biomolecules.

**Figure 1:**
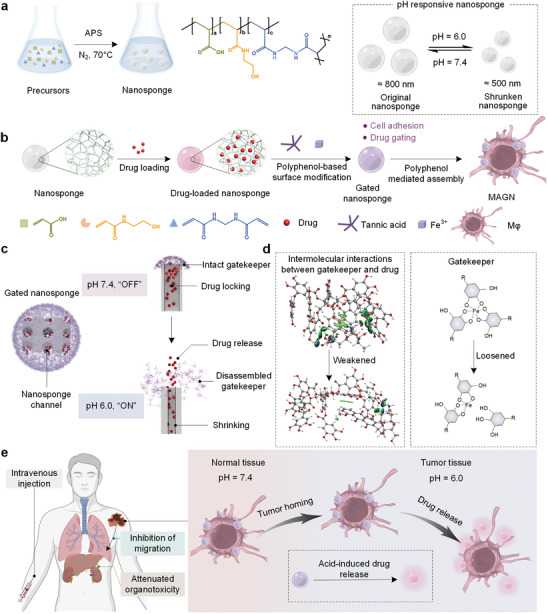
Stimuli‐responsive nanosponges capped with metal‐phenolic supramolecular gatekeepers anchored on tumor‐targeting M*φ* (MAGN platform) against metastatic tumors. a) Synthesis of pH‐responsive nanosponges by free‐radical polymerization. b) Schematic illustration of the nanosponges being loaded with a chemotherapeutic drug (doxorubicin, DOX) and attached to M*φ* via cell surface engineering. c) Blocked drug diffusion due to the intermolecular interactions between the metal‐phenolic supramolecular gatekeepers and drugs. d) Mechanism of the accelerated drug release within the acid tumor microenvironment. e) Evaluation of the antitumor effect with MAGN.

Herein, we report a class of macrophage‐driven automated homing of gated nanosponges, referred to as MAGN, in which biologically responsive supramolecular‐gated porous nanogels (namely “nanosponge”) can be integrated on the surfaces of tumor‐homing macrophages (M*φ*s).^[^ ^13^, ^29^ ^]^ We demonstrate that a high content of therapeutic drugs can be robustly encapsulated in nanosponges capped with metal‐phenolic supramolecular gatekeepers (**Figure** **1**a, b). Owing to their three dimensional (3D) coordination networks, the channels of the nanosponges can be efficiently blocked by the iron‐tannic acid (Fe^III^‐TA) supramolecular networks.^[^ ^30^, ^31^ ^]^ Subsequent adhesion of the galloyl and catechol units of TA onto the cell surfaces mediates the assembly of drug‐loaded nanosponges on the bioactive M*φ* (Figure 1b, c).^[^ ^32^, ^33^ ^]^ Upon exposure to acidic tumor microenvironments, the disassembly of the Fe^III^‐TA gatekeeper and the significant shrinking of nanosponges lead to the controlled release of drugs in targeted sites (Figure 1d). Our in vivo results show the site‐specific accumulation of MAGN in metastatic melanoma, which resulted in a systemic decrease in tumor burden and lung metastases (Figure 1e). These findings highlight the benefits of combining cellular surface engineering with supramolecular‐gated porous nanosponges and enable a unique tumor‐targeted drug delivery system for the effective treatment of metastatic cancers.

## 2 Results and Discussion

**Figure 2:**
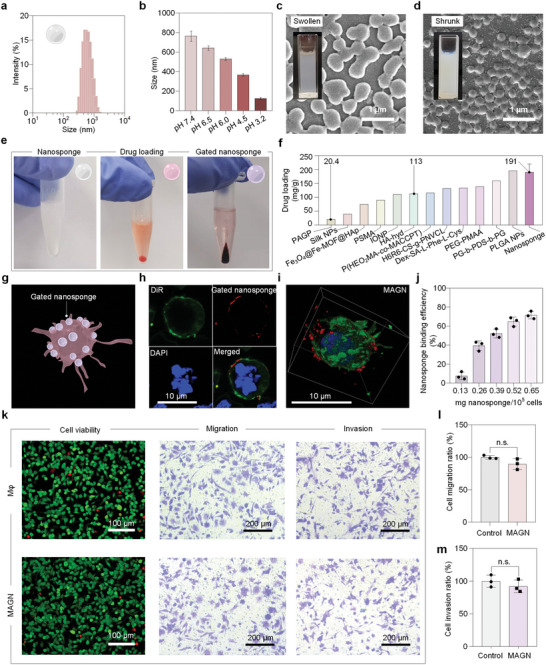
Characterization and drug loading of nanosponges and their binding to the surface of engineered M*φ*. a) Size distribution of nanosponges. b) Average size of nanosponges at different pH values. c, d) SEM images and photographs (inserted) of swollen (in an alkaline medium, pH 9.0) and shrunk (in an acidic medium, pH 4.0) nanosponges. Scale bars, 1 µm. e) Photographs of nanosponges, drug‐loaded nanosponges, and gated nanosponges in Eppendorf tubes. f) Comparison of the maximum drug loading of nanosponges and other carriers.^[^ ^34^, ^35^, ^36^, ^37^, ^38^, ^39^, ^40^, ^41^, ^42^, ^43^, ^44^, ^45^, ^46^ ^]^ g) Schematic illustration of MAGN. h) Confocal micrographs of M*φ*s (cell nucleus, blue; membrane, green) displaying nanosponge with DOX (red). Scale bars, 10 µm. i) 3D confocal micrographs of MAGN. Scale bar, 10 µm. j) Binding efficiency of nanosponges to M*φ* as a function of nanosponge concentration. k) Cell function evaluation with fluorescence microscopy (green, live cells; red, dead cells) and transwell assay for M*φ* and MAGN. Scale bars, 100 and 200 µm. l, m) Percentages of M*φ* and MAGN that migrated and invaded, normalized to untreated M*φ* (control). n.s., not significant.

We first characterized the morphology and size of the pH‐responsive nanosponges, which were free‐radical polymerized from N‐(2‐Hydroxyethyl) acrylamide (HEAA) and acrylic acid (AA) as the monomers and N, N'‐methylenebis(acrylamide) (MBA) and ammonium persulfate (APS) as the crosslinker and initiator, respectively. As shown in **Figure** **2**a, the average diameter of the nanosponges determined by dynamic light scattering (DLS) was 765 nm, which was further confirmed by scanning electron microscope (SEM) (Figure S1, Supporting Information) and transmission electron microscope (TEM) (Figure S2, Supporting Information). Then, the pH‐triggered‐shrinking capability of the nanosponges was evaluated by DLS at different pH values in the range from 7.4 to 3.2, showing the diameter of the nanosponges gradually reduced from ≈800 to ≈200 nm (Figure 2b and Table S1, Supporting Information), which was also confirmed by SEM images. Visually, the nanosponges in an alkaline medium was nearly transparent, while in an acidic medium, the nanosponges solution became more turbid (Figure 2c, d), which could be attributed to the ionizable carboxyl acid groups in nanosponges that could accept and donate protons in response to a variation of pH. Typically, carboxyl acid is deprotonated above its *p*K_a_, leading to a high network osmotic pressure and hence to the expansion of the nanosponges. Conversely, carboxyl acid is protonated below its *p*K_a_, which drives the collapse of the nanosponges and the water being squeezed out. Subsequently, the pH‐responsive nanosponges were shown to encapsulate a high mass of drugs, using doxorubicin (DOX) as a model chemotherapeutic drug. As shown in Figure 2e, after the diffusion equilibrium of drug into the nanosponges was reached, the nanosponges were centrifugally separated to produce a red precipitate with a high‐efficiency drug loading (191 ± 29 mg g^−1^, up to 22 wt.%, Figure 2f) due to the high specific surface area and internal network structure of the nanosponges, indicating the successful loading of drug. To gate (i.e., stably sequester) drugs inside the nanosponges and robustly attach the nanosponges to cells, the surfaces of the drug‐loaded nanosponges were modified with metal‐phenolic coatings by tannic acid (TA) and Fe^III^ ions. Fe signal from elemental analysis of the gated porous nanosponges indicated the formation of the metal‐phenolic nanogatekeepers on the drug‐loaded nanosponges (Figure S3, Supporting Information).

To facilitate stronger interactions with the nanosponges, M*φ*s were pretreated with cationic cellulose to adjust the zeta potential of M*φ*s from −14.7 to 16.0 mV (Figure S4, Supporting Information). Then, the gated nanosponges and the pretreated M*φ*s were rapidly mixed for 30 s to obtain the nanosponges engineered M*φ*s (referred to as a MAGN platform). Confocal microscopy images showed that the nanosponges (red) were homogeneously decorated on the outer membranes of M*φ* (Figure 2g–i). In addition, nanosponges still remained on the surface of M*φ* after incubation for 12 h, which is likely due to the relatively large size of nanosponges (over 700 nm under pH 7.4 condition) that prolong the phagocytosis of M*φ* during the attachment and drug delivery process (Figure S5, Supporting Information). Next, the binding efficiency of the nanosponges to M*φ* was measured by flow cytometry (Figure 2j). With an increasing concentration of incubated nanosponges, the binding efficiency between nanosponges and M*φ* was increased. Particularly, 71% of M*φ*s were found to carry nanosponges, when incubated with 0.65 mg of nanosponges per 10^5^ cells. Benefiting from such high binding efficiencies and the high drug loading capacity of the nanosponges, M*φ*s were able to carry a high dose of DOX (as high as 88.6 µg per 10^5^ M*φ*s) (Figure S6, Supporting Information). To evaluate whether the high content of chemotherapeutic drug affects the normal functions of M*φ* (i.e., viability, migration, and invasion), the M*φ*s with 71% assembly efficiency group were studied in a transwell setup. From Figure 2k, the cell viability of the M*φ*s with 71% assembly efficiency was not significantly affected after 4 h, indicating that the surface engineering process and the encapsulated chemotherapeutic drug showed no toxicity to the cell vehicle at the pH of normal tissue (7.4). In addition, the delivery of cargo relies on the migration and invasion of the M*φ*. In this study, we evaluated the migration and invasion ability of the engineered M*φ* without or with a matrigel layer, respectively. Both results indicated that there was no significant effect on the migration and invasion ability of the engineered M*φ* by the attachment of nanosponges compared with the control group after 24 h (M*φ* alone) (Figure 2k–m). In addition, the cell viability of M*φ* in MAGN after incubation for 12 h still remained at ≈71% as the control group (Figure S7, Supporting Information). These results motivated the further evaluation of this cell‐based drug delivery system in vivo.

**Figure 3:**
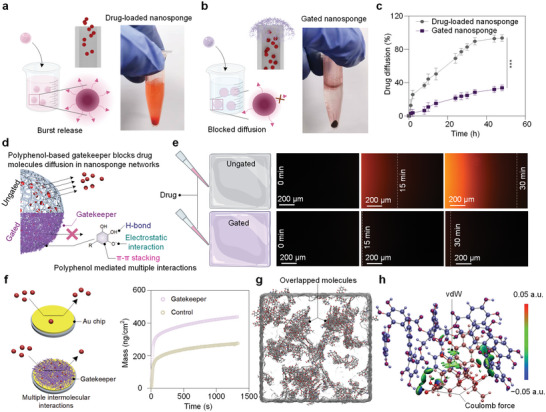
Encapsulation of drugs in nanosponges capped by Fe^III^‐TA gatekeeper. a, b) Drug diffusion from ungated nanosponges and gated nanosponges after being incubated in PBS at pH 7.4 for 30 min. c) Time‐dependent diffusion of chemotherapeutic drug from ungated and gated nanosponges. All data represent the mean ± SD (n =3), and the statistical significance was calculated via one way ANOVA with Tukey's multiple comparisons (*p<0.05, **p<0.01, ***p<0.001). d) Schematic illustration of the Fe^III^‐TA gatekeeper blocking drug diffusion out of the nanosponges. e) Diffusion of the drugs in ungated and gated hydrogel sheets observed by fluorescence microscopy. Scale bar, 200 µm. f) Schematic representation of the Fe^III^‐TA gatekeeper on QCM chips, which enhanced the mass adsorption of drug due to the multiple interactions between polyphenol moieties and model drug DOX. The change of interfacial interactions can be detected by the frequency change, *Δf*, which is proportional to the mass of the absorbed molecules, *Δm*. QCM results show the mass change over time as chemotherapeutic drug flowed over a bare gold substrate or a substrate coated with Fe^III^‐TA gatekeeper. g) Equilibrium state of the simulation boxes packed with 20 TA molecules and 80 DOX molecules at pH 7.4. h) Equilibrium molecular interaction energies between TA and DOX in pH 7.4 solution.

To gain insight into the interactions between the polyphenol‐based gatekeeper and drug molecules, drug diffusion, and quartz crystal microbalance (QCM) studies were employed to investigate the adsorption of drug molecules on the Fe^III^‐TA gatekeeper. As shown in **Figure** **3**a, nanosponges ungated by Fe^III^‐TA networks released chemotherapeutic drugs through a burst release, showing a distinctly orange color in solution after 30 min. Contrastly, nanosponges modified with Fe^III^‐TA networks to form gatekeepers blocked the diffusion of drug, resulting in a relatively clear solution after 30 min (Figure 3b). The cumulative release profile of DOX also illustrated that the concentrations of drug released from the original nanosponges were significantly higher than that from the gated nanosponges (Figure 3c). Specifically, after incubating drug‐loaded nanosponges in 1 mL of water for 48 h, over 80% of the loaded drug was released, while only ≈37% of the loaded drug was released from the gated nanosponges after 48 h at pH 7.4 (Figure 3d). To better visualize the reduction in speed of drug release by the addition of the Fe^III^‐TA gatekeeper, a linear diffusion experiment was carried out over 30 min. The directional diffusion of fluorescent drug in the gated nanosponges (< 0.55 µm s^−1^) was substantially slower than that of the ungated nanosponges (5.55 µm s^−1^) (Figure 3e, Videos S1, and S2, Supporting Information), indicating that the Fe^III^‐TA gatekeepers provide a remarkable (10 ×) effect on suppressing the diffusion of drug. In addition, QCM was used to evaluate the interactions between the loaded drugs and the Fe^III^‐TA gatekeeper directly (Figure 3f). A small amount of the adsorption mass (≈250 ng cm^−2^) was detected on a pure Au chip. In contrast, the adsorption mass on the Au chip covered with Fe^III^‐TA gatekeeper increased significantly (≈430 ng cm^−2^) after the addition of drugs, indicating strong interactions between the drug and the Fe^III^‐TA gatekeeper. These results revealed that the Fe^III^‐TA gatekeeper of the nanosponges could greatly suppress the diffusion of chemotherapeutic drugs loaded within the nanosponges.

To understand the mechanisms for how the Fe^III^‐TA gatekeeper slows the rate of drug release, we investigated the molecular interactions between the gatekeeper and model drug DOX by molecular dynamics (MD) simulations and density functional theory (DFT) calculations. The initial simulation boxes had dimensions of 150 × 150 × 150 Å^3^ and were packed with 20 TA molecules, 80 DOX molecules, and 20000 water molecules using the packmol program.^[^ ^47^ ^]^ In order to differentiate the different molecules, the atoms were marked with different colors (as shown in Figure 3g, C:black, O:red, N:blue). When the system reached equilibration, the TA and DOX molecules were closely overlapped compared to their start states, indicating strong interactions formed between TA and DOX molecules (Figure 3g; Figure S8, Supporting Information). The particle‐mesh Ewald (PME) method with a cut‐off distance of 20 Å was applied to treat the Coulomb force and the van der Waals forces (vdW). Due to the abundance of phenolic hydroxyl groups, Coulombic interactions were found to be the main intermolecular interaction between TA and DOX, which has a positive charge across the surface of the DOX molecules (the elliptic balls in Figure 3h, −0.992 eV). In addition, vdW interactions from the benzene rings in both DOX and TA molecules were found to play an inferior role in the intermolecular interactions (the green schistose in Figure 3h, −0.627 eV).

**Figure 4:**
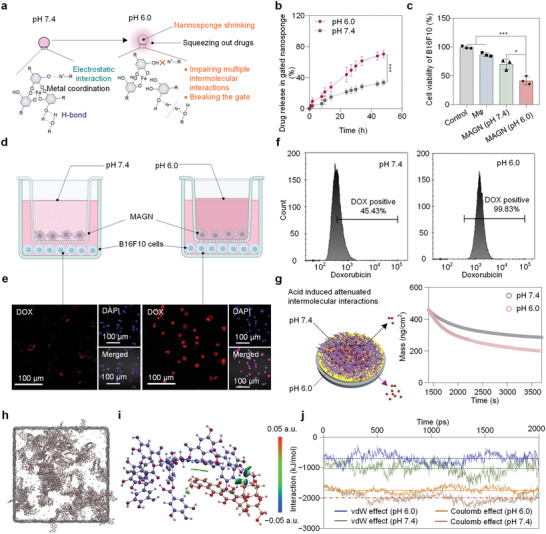
Molecular behavior and mechanistic study of stimuli‐responsive drug release. a) Schematic representation of chemotherapeutic drug release under an acidic environment. b) Time‐dependent release of chemotherapeutic drug (DOX) from gated nanosponges at pH 6.0 and 7.4, respectively. All data represent the mean ± SD (n = 3), and the statistical significance was calculated via one way ANOVA with Tukey's multiple comparisons (***p<0.001). c) B16F10 cell viability under different culture conditions for 24 h. d) Schematic illustration of co‐cultures of MAGN and B16F10 cells in different pH conditions. e, f) CLSM and flow cytometry showing the chemotherapeutic drug content of treated B16F10 cells under different conditions. For CLSM, cell nuclei were stained using 4′,6‐diamidino‐2‐phenylindole (DAPI). Scale bars, 100 µm. g) Schematic representation of QCM chips coated with Fe^III^‐TA gatekeeper and washed by PBS at different pH values after the absorption of chemotherapeutic drug molecules. QCM analysis shows a change in mass over time as different pH solutions flowed over the substrate. h) Simulation boxes packed with 20 protonated TA molecules and 80 protonated DOX molecules by MD simulation in the equilibrium pH 6.0 state. i) Equilibrium molecular interaction energy of protonated TA molecules with protonated DOX molecules in pH 6.0 solution. j) MD simulation plots for the interaction of DOX and TA molecules against time at equilibrium.

To verify the on‐demand release of drug from the gated nanosponges in the acidic microenvironment of solid tumors, we assayed the release profile of DOX as a model chemotherapeutic drug in pH 6.0 and pH 7.4 media, which mimicked the microenvironment of the tumor and the circulatory system, respectively (**Figure** **4**a). As shown in Figure 4b, the gated nanosponges generally exhibited a faster release of chemotherapeutic drug (> 44%) at pH 6.0 compared to the cumulative release of chemotherapeutic drug (< 25%) at pH 7.4 after 24 h, which likely results from the dissociation of polyphenol‐based gatekeeper and the shrinkage of nanosponges at pH 6.0.

Cell viability tests at different pH conditions were carried out to investigate the capability of the MAGN to kill B16F10 cells in the acidic tumor microenvironment (Figure 4c). Compared to the control group, the viability of B16F10 cells treated with MAGN at pH 7.4 and pH 6.0 media were 64% and 44%, respectively, suggesting that MAGN is more effective at killing B16F10 cells under an acidic environment. To further investigate the concentration of chemotherapeutic drugs in B16F10 cells visually, the uptake of drug by the B16F10 cells was tested by a laser scanning confocal microscope (CLSM) and flow cytometry. The fluorescence intensity of B16F10 cells treated with MAGN at pH 6.0 was significantly higher than that of the cells treated with MAGN at pH 7.4, indicating that more chemotherapeutic drug was released from MAGN at the simulated acidic environment (Figure 4d, e), which was consistent with the fluorescence intensity of cells (Figure S9, Supporting Information). Meanwhile, the number of B16F10 cells in the positive chemotherapeutic drug reached 45% after incubation with MAGN at pH 7.4 for 2 h, whereas that same group reached 99% after incubation at pH 6.0 for 2 h (Figure 4f), suggesting again that drug release from the MAGN will be enhanced in the acidic microenvironment of solid tumors.

We further explored the reasons for these differences at pH 7.4 and pH 6.0. As shown in Figure S10 (Supporting Information), the ungated nanosponges were placed in different pH environments, and the accumulative release of chemotherapeutic drugs from the nanosponges at pH 6.0 was much higher than that at pH 7.4, which was supported by the UV–vis spectrum peak at 482 nm of its supernatant. This could be attributed to the fact that swollen nanosponges dispersed in pH 7.4 switch to a shrunken state in acidic environments due to the protonation of carboxylic acid groups, thereby subsequently expelling chemotherapeutic drug molecules. In addition, QCM was used to evaluate the intermolecular interactions of chemotherapeutic drug and Fe^III^‐TA gatekeeper under an acidic environment. It was found that after the Fe^III^‐TA gatekeeper adsorbed chemotherapeutic drug to equilibrium, washing the Fe^III^‐TA gatekeeper covered Au chips with PBS at different pH (Figure 4g), more chemotherapeutic drugs were washed away at pH 6.0 compared to that at pH 7.4. Collectively, these data suggest that the pH plays a deterministic role in regulating the intermolecular interactions between chemotherapeutic drugs and the Fe^III^‐TA gatekeeper. To further understand the changes of molecular interactions at pH 6.0, 20 protonated TA molecules and 80 protonated DOX molecules were constructed by MD simulations (Figures S11 and S12, Supporting Information). When these molecules reached the equilibrium state in the pH 6.0 solution, the looser bonding of DOX and TA in Figure 4h indicated a weaker interaction compared to those in Figure 3h (C:black, O:red, N:blue). The energy between TA and DOX molecules was calculated by DFT calculations, where the Coulomb interaction was −0.383 eV, and the vdW interaction was −0.331 eV (Figure 4i, the carbon atoms on TA and DOX molecules were labeled blue and red, respectively). In addition, the variation function of the intermolecular interaction energy with time showed that the vdW and Coulomb interactions at pH 6.0 were significantly weaker than those at pH 7.4 (Figure 4j). These results confirm that the intermolecular interactions between the gatekeeper and the drug are weaker in acidic environments (pH 6.0) than that in pH 7.4, which jointly lead to controlled drug release in the acidic tumor microenvironment.

**Figure 5:**
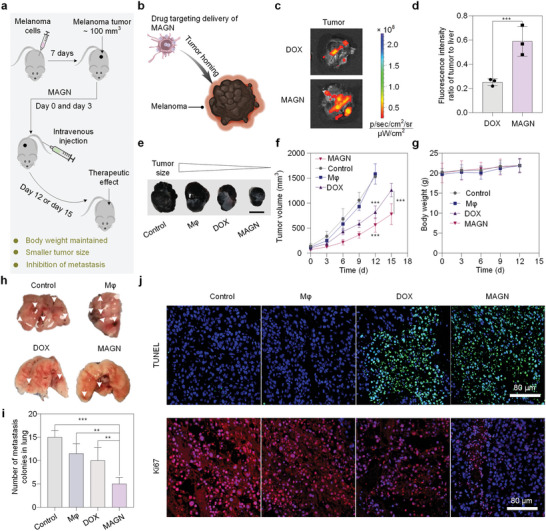
MAGN platform leads to significant suppression of melanoma tumor growth and lung metastasis. a) Schematic representation of tumor inoculation and treatment. b) In vivo tumor targeting of MAGN evaluated in mice bearing B16F10 tumors. c) In vivo imaging system (IVIS) images of tumors 24 h after i.v. injection of MAGN and free chemotherapeutic drug. d) Fluorescence intensity ratios of the tumor to the liver for free chemotherapeutic drug and MAGN groups. All data represent the mean ± SD (n = 3), and the statistical significance was calculated via one way ANOVA with Tukey's multiple comparisons (***p<0.001) e) Images of tumors after the final treatment. Scale bar, 1 cm. f) Tumor growth profiles after treatment with different formulations. g) Plot of body weight versus time in tumor‐bearing mice. h) Morphological changes in lungs at day 12 or 15. i) Number of visible metastatic nodules on the lungs for different groups. j) Histological analysis of tumor section stained with TUNEL and Ki67 for mice with different treatment groups stained with DAPI (blue), TUNEL (green), and Ki67 (red). Scale bars, 80 µm.

The antitumor activity of MAGN was evaluated in C57BL/6J mice bearing B16F10 melanomas (**Figure** **5**a), in which M*φ* could actively target the melanoma due to their specific tumor‐homing properties.^[^ ^48^ ^]^ B16F10 tumor‐bearing mice were divided randomly into two groups and then i.v. injected with the free chemotherapeutic drug and MAGN cells, respectively (n = 3). Thereafter, the chemotherapeutic drug concentration at the tumor sites was measured by a live imager after 24 h (Figure 5b, c). The mice treated with MAGN showed a significantly higher fluorescence intensity (≈3×) compared to the mice treated with free chemotherapeutic drug, which supported our hypothesis that MAGN can actively target tumor sites (Figure 5d). Meanwhile, the gated nanosponges showed negligible tumortargeting ability compared with chemotherapeutic drug (Figure S13, Supporting Information). In addition, we found that the majority of the chemotherapeutic drug was initially accumulated in the liver (Figures S14 and S15, Supporting Information), which was consistent with many drug delivery systems and could be attributed to the first­pass effect.^[^ ^7^ ^]^

Antitumor effects of MAGN in vivo were evaluated in the mice bearing B16F10 subcutaneous tumors (≈500 mm^3^). Mice were randomly sorted to receive the following treatments (n = 5 per group) via i.v. injection on day 0 and day 3 for two doses: i) PBS; ii) free chemotherapeutic drug (3 mg DOX kg^−1^); iii) gated nanosponges (3 mg DOX kg^−1^); iv) M*φ* (2 × 10^6^ cells per mouse); and v) MAGN (3 mg DOX kg^−1^, ≈2 × 10^6^ cells per mouse). The efficacy of the antitumor treatment was evaluated by monitoring tumor growth and body weight for 15 days. Compared to the PBS and M*φ* treatment groups, the chemotherapeutic drug, gated nanosponges, and MAGN treatment groups exhibited an effective reduction of tumor growth, which was consistent with our cell toxicity results in vitro (Figure 5e; Figures S16 and S17, Supporting Information). For the free chemotherapeutic drug and MAGN groups, we observed significant tumor suppression by day 12 (groups treated with PBS and M*φ* were sacrificed prematurely due to the large size of the tumors) (Figure 5f; Figure S18a, Supporting Information). For the free chemotherapeutic drug groups, the average tumor volumes were 1261 mm^3^ on day 15. Relative to the PBS group, the tumor growth inhibition (TGI) rate (the ratio of the tumor volume reduced by the experimental group relative to the tumor volume of the control group) was 29.9% on day 15. As a comparison, the MAGN group showed an impressive TGI rate of 45.2% on day 15, which indicated that the targeted delivery of MAGN significantly inhibited tumor growth compared to free chemotherapeutic drugs. No significant body weight changes were observed in the MAGN group compared to the PBS group during the whole treatment process, indicating the safety of the MAGN in tumor treatment (Figure 5g; Figure S18b, Supporting Information). We then examined the potential of MAGN to inhibit the metastatic spreading of melanoma to the lungs. The group treated with MAGN led to significantly fewer metastatic nodules in the lung than the other groups (Figure 5h, i). These results indicate that MAGN has the potential to treat not only the primary tumor, but also suppress tumor metastasis.

To quantify apoptosis and proliferation of tumor cells, excised tumors were stained with terminal deoxynucleotidyl transferase‐mediated dUTP nick‐end labeling (TUNEL) and Ki67. The highest positive TUNEL signals and the weakest Ki67 signals of MAGN suggested that MAGN inhibited tumor cell proliferation. The most severe morphological change and cell death from tumor slices were observed in tumors treated with MAGN, indicating that MAGN showed the strongest antiproliferative effects over these four groups studied (Figure 5j). We also examined blood samples obtained from treated mice. For the MAGN group, aspartate aminotransferase (AST), and kidney function (Creatinine) were within normal physiological ranges (Figure S19, Supporting Information). Moreover, to further evaluate the safety of MAGN, we conducted the blood routine examination and H&E‐stained tissue sections of the heart, liver, spleen, lung, and kidney of the mice on day 15, no significant difference in blood and histological changes were observed in both control and treatment groups (Figures S20, S21, and Table S2, Supporting Information).

## 3 Conclusion

We constructed a cell‐based drug delivery system to enhance antitumor efficacy by engineering tumor‐homing M*φ* decorated with supramolecular‐gated nanosponges, named MAGN. The rationally designed nanosponges are able to drastically shrink in an acidic environment, leading to the release of encapsulated chemotherapeutic drugs. The highly porous and hydrophilic characteristics of the nanosponges allowed for high‐efficiency loading of drugs (up to 22 wt.%). The polyphenol‐based supramolecular gatekeepers endowed the nanosponges with the ability to attach to cellular surfaces, and the “on‐off” gating ability of the Fe^III^‐TA gatekeeper enabled controllable drug release. Our mechanistic studies revealed that the Fe^III^‐TA gatekeeper can inhibit drug diffusion via strong intermolecular Coulombic forces and vdW interactions during their transport at pH 7.4. Once the nanosponges arrive in an acidic environment, their intermolecular interactions are significantly reduced, allowing drug release locally in the tumor microenvironment. The MAGN platform successfully delivered the model chemotherapeutic drug to melanoma tumors with an efficiency that was 3 times higher than the free drug alone, which enabled systemic tumor suppression to resist the formation of new lung metastases. Due to the highly porous structure of the designed nanosponges, the MAGN platform may serve as a versatile platform to achieve high loading and enhanced delivery of various therapeutic drugs (e.g., DNA/RNA, monoclonal antibodies, and peptides) to the desired pathological site.

## Conflict of Interest

The authors declare no conflict of interest.

## Author Contributions

X.L. and G.G. contributed equally to this work. J.G., X.L., J.S., and G.G. conceived the project. X.L., J.S., and G.G. designed the experiments and performed the most experiments and data analyses. M.D., Z.X., and X. H. assisted with the animal experiments. J.G., X.L., J.S., G.G., Z.Z., A.M.B., and C.W.S. wrote and reviewed the manuscript. All the authors discussed the results and commented on the manuscript.

## Supporting information

Supporting Information

Supplemental Video 1

Supplemental Video 2

## Data Availability

The data that support the findings of this study are available from the corresponding author upon reasonable request.
