# Supplementary material for: Systemic Tumor Suppression via Macrophage‐Driven Automated Homing of Metal‐Phenolic‐Gated Nanosponges for Metastatic Melanoma
Source: Adv Sci (Weinh). 2023 Apr 18;10(18):2207488. doi: 10.1002/advs.202207488 (PMC10288275; doi:10.1002/advs.202207488)
Supplement: Supplementary file 1 — Supporting Information [file ADVS-10-2207488-s003.pdf]

ADVANCED SCIENCE
Open Access

Supporting Information

for *Adv. Sci.*, DOI 10.1002/advs.202207488

Systemic Tumor Suppression via Macrophage-Driven Automated Homing of Metal-Phenolic-Gated Nanosponges for Metastatic Melanoma

Xue Liao, Guidong Gong, Mengyuan Dai, Zhenyu Xiang, Jiezhou Pan, Xianglian He, Jiaojiao Shang, Anna Maria Blocki, Zongmin Zhao, C. Wyatt Shields IV and Junling Guo**

Supporting Information

Systemic Tumor Suppression via Macrophage-Driven Automated Homing of Metal-Phenolic-Gated Nanosponges for Metastatic Melanoma

Xue Liao, Guidong Gong, Mengyuan Dai, Zhenyu Xiang, Jiezhou Pan, Xianglian He, Jiaojiao Shang*, Anna Maria Blocki, Zongmin Zhao, C. Wyatt Shields IV, and Junling Guo*

Table of Contents

Section S1. General materials 3
Section S2. Instruments 3
Section S3. Synthesis of nanosponges 4
Section S4. Synthesis of drug-loaded nanosponges 4
Section S5. Synthesis of metal-phenolic supramolecular-gated nanosponges 4
Section S6. *In vitro* drug release study 5
Section S7. Drug diffusion 5
Section S8. QCM experiments 5
Section S9. Biological studies 5
Section S10. Binding gated nanosponges to macrophages 6
Section S11. Confocal laser scanning microscopy (CLSM) observation 6
Section S12. Live/Dead cell staining 6
Section S13. MAGN viability assay 7
Section S14. Cell migration/invasion assay 7
Section S15. Interaction of drug released from MAGN with B16F10 cells 7
Section S16. Toxicity of MAGN to B16F10 melanoma cells 8
Section S17. Cell viability assay 8
Section S18. *Ex vivo* biodistribution assay 9
Section S19. *In vivo* therapy study 9
Section S20. Supplementary figures S1–S21 10
Section S21. Supplementary tables S1–S2 31
Section S22. Supplementary references 33

Section S1. General materials

N-(2-Hydroxyethyl) acrylamide (HEAA, 98%), N, N'-methylenebis(acrylamide) (MBA, ≥99.5%), ammonium persulfate (APS, 99%), sodium dodecyl sulfate (SDS, ≥99%), polyquaternium-10 (RG, 99%), guar gum and gelatin were purchased from Titan Technology Co., Ltd (Shanghai, China). Acrylic acid (AA, 99.5%) was obtained from Aladdin Chemistry Co., Ltd (Shanghai, China) and was purified by a neutral aluminum oxide column before use. Tannic acid (TA, 95%) was purchased from Sigma-Aldrich (USA). Ferric (III) chloride hexahydrate ($FeCl_3 \cdot 6H_2O$, ≥99.0%) was purchased from Chron Chemical Co., LTD (Shanghai, China). Doxorubicin hydrochloride (DOX·HCl, 98%) was purchased from Aladdin (Shanghai, China). PBS buffer was purchased from Adamas life (China). All reactants were of analytical purity grade and were used as received without further purification. 6-well plates, 24-well plates, and 96-well plates were supplied by Jetbiofil Cnreagent Co., Ltd (Guangzhou, China). Dulbecco's Modified Eagle Medium (DMEM), Roswell Park Memorial Institute 1640 (RPMI 1640), Fetal Bovine Serum (FBS), penicillin-streptomycin, Trypsin-EDTA solution (0.25%), giemsa stain, 4,6-diamidino-2-phenylindole (DAPI) and 1,1-dioctadecyl-3,3,3,3-tetramethylindotricarbocyanine iodide (DiR) were purchased from Sigma-Aldrich (USA). Cell Counting Kit-8 was sourced from KGI Biotechnology Co., Ltd (Jiangsu, China). Calcein-AM/EthD-I was provided by Biyuntian Biotechnology Co., Ltd (Shanghai, China). High-purity Milli-Q (MQ) water with a resistivity of 18.2 MΩ cm was obtained from an inline Millipore RiOs/Origin water purification system. All solutions were freshly prepared for immediate use in each experiment.

Section S2. Instruments

Scanning electron microscopy (SEM) was performed on a JEOL JSM-7500F SEM instrument. Transmission electron microscopy (TEM) was performed on a Tecnai G2 F20 S-TWIN TEM instrument, operating at a voltage of 100 kV (FEI USA, Inc.). Size distribution of the particles were measured by DLS on Zetasizer Nano ZSP (Malvern, UK). The aqueous solutions were prepared at certain concentration, at least three measurements were made for each sample. The results are expressed as the mean and standard deviation obtained from three measurements. UV-Visible absorption measurements were conducted on a Lambda 950 spectrophotometer. Fluorescence microscope images were obtained using an inverted Olympus CKX53 microscope. The confocal laser scanning microscopy (CLSM) were obtained on an FV1000 confocal laser-scanning microscope (Olympus, Tokyo, Japan). CCK-8 assay was performed with an Infinite nano microplate reader (Tecan Group, Switzerland). Flow cytometry assays were performed on an Exflow-104 flow cytometer (DAKEWE, China). In vivo imaging experiments were performed with IVIS spectrum small-animal imaging system (IVIS Lumina Series III, PerkinElmer). High angle angular dark field-scanning transmission

electron microscopy (HAADF-STEM) was performed on a Talos F200S TEM instrument.

Section S3. Synthesis of nanosponges

1 g (8.7 mmol) of HEAA and 88 mg (1 mmol) of AA were dissolved in 45 mL MQ water, then 79 mg (0.5 mmol) of MBA and 22 mg of SDS (0.05 wt%) were added to the solution. Subsequently, the solution was purged with nitrogen at least 30 min to remove oxygen under stirring, and then heated up to 70 °C. Finally, APS (28 mg, 10 mg/mL) solution was added into the preheated solution to initiate and proceed the polymerization under N_2 atmosphere for 0.5 h. The resultant nanosponges dispersion was dialyzed (MWCO = 3000) against water for 3 days to remove unreacted monomers and other small molecules. After dialysis, the nanosponges were lyophilized and stored in a vacuum desiccator at room temperature.

Section S4. Synthesis of drug-loaded nanosponges

5.2 mg of dry nanosponges and 2.6 mg of DOX were dispersed in 2.6 mL of MQ water under stirring for 10 min at room temperature. The dispersion was then centrifugated to collect the sediments of DOX-loaded nanosponges and washed for three times with deionized water to remove the surface adsorbed DOX. The purified DOX-loaded nanosponges were freeze-dried and stored in a vacuum desiccator at room temperature for the further experiments.

Section S5. Synthesis of metal-phenolic supramolecular-gated nanosponges

5.2 mg of dry drug-loaded nanosponges were dispersed in 1 mL of phosphate buffer solution (pH 7.4, 10 mM), then deposition of the metal-phenolic supramolecular on nanosponges was conducted by sequential addition of 7 μL of TA solution (10 mg/mL) and 7 μL of $FeCl_3{\cdot}6H_2O$ solution (10 mg/mL). The solution mixture was vigorously vortexed for 10 s. The resulting suspension was washed with MQ water, the above deposition step was repeated once, then the sediment was washed three more times and redispersed in 1 mL of MQ water. The DOX mass loaded in nanosponges was calculated by subtracting the mass of DOX in the total supernatant from the total mass of the drug in the initial solution by a UV-visible spectrophotometer at 480 nm (comparing with DOX calibration curve). The resulting Fe^{III}-TA supramolecular networks gated nanosponges were used for the further experiment. During the storage of gated nanosponge, we noticed that the gated nanosponges were more stable in MQ water than in isotonic solutions.

The drug loading content (DLC) was calculated according the following equations:

DLC (%, DOX) = (initial weight of DOX − weight of DOX supernatant) / weight

of drug loaded nanosponges × 100%

Section S6. *In vitro* drug release study

Gated nanosponges with drugs were resuspended in 1 mL complete medium (pH 7.4 and pH 6.0, respectively) and shaked at 37 °C in a tube. At regular time points, the nanosponges suspension was centrifuged at 8800 g for 4 min and the supernatant was collected for analysis, and the nanosponges were further resuspended in 1 mL of fresh release media and shaked at 37 °C until the next time point. The supernatant samples were taken at 1, 2, 6, 10, 20 and 24 h after shaking. The cumulative release was quantified by a UV-visible spectrophotometer at 480 nm (comparing with DOX calibration curve).

Section S7. Drug diffusion

Aldehydated guar gum and gelatin were cross-linked to be hydrogels by Schiff base reaction. For the control group, 10 μL of hydrogel solution was dropped on the glass slide covered with a cover glass to be a hydrogel sheet with the size of 1 cm × 1 cm × 0.1 cm. And then DOX solution (0.1 mg/mL, 5 μL) was continuously added to one side of the cover glass for the detection of drug diffusion rate. For the experimental group, 100 μL of TA solution (10 mg/mL) and 100 μL of $FeCl_3·6H_2O$ (10 mg/mL) were added to 1 mL of hydrogel solution with intensely mixing, and then 10 μL of mixed solution was added to the glass slides for hydrogel sample preparation. Finally, DOX solution was continuously added to one side of the cover glass. Video and photo shoots were performed for watching the drug diffusion visually.

Section S8. QCM experiments

The amount of DOX captured by the Fe^{III}-TA gatekeeper was compared through Fe^{III}-TA supramolecular networks covered Au chip or free Au chip, then flowing a solution of the appropriate DOX (0.1 mg/mL) over these Au chips and evaluating absorbed mass using a QSense Explorer (Biolin Scientific AB, Sweden) quartz crystal microbalance. After the equilibrium of DOX adsorption, the Fe^{III}-TA deposited Au chip groups were washed with pH 7.4 and 6.0 PBS buffer (10 mM), respectively. The dynamic tests were carried out at a constant flow rate of 75 μL/min. The mass of the immobilized DOX was calculated by the Sauerbrey model.

Section S9. Biological studies

B16F10 cells were maintained in Dulbecco's Modified Eagle Medium (DMEM) containing 10% fetal calf serum and 1% penicillin/streptomycin at 37 °C in a 5% CO_2

atmosphere. RAW264.7 cells were placed in Roswell Park Memorial Institute 1640 (RPMI 1640) containing 10% fetal calf serum and 1% penicillin/streptomycin at 37 °C in a 5% CO_2 atmosphere.

Section S10. Binding gated nanosponges to macrophages

Macrophages （1×10^6 cells） were placed into 1mL PBS buffer (pH 7.4, 100 mM), and supplemented with 10 μL cationic cellulose (polyquaternium-10, 30 mg/mL) and mixed for 30 s to make the cell surface positively charged. Then, the macrophages were washed three times with PBS buffer to remove excess polyquaternium-10. 0.13, 0.26, 0.39, 0.52 or 0.65 mg of Fe^{III}-TA supramolecular networks gated nanosponges were added to the 1 mL treated macrophages and mixed for 30 s. The resulting suspension was washed three times with PBS buffer, and then redispersed in DMEM medium. The resulting macrophages loaded with gated nanosponges were referred to as the MAGN to be confirmed by confocal microscopy and used in the subsequent studies.

Section S11. Confocal laser scanning microscopy (CLSM) observation

To utilize confocal imaging, macrophages nucleus and membrane were labeled with the fluorescent dye 4,6-diamidino-2-phenylindole (DAPI) and 1,1-dioctadecyl-3,3,3,3-tetramethylindotricarbocyanine iodide (DiR), respectively. For *in vitro* investigations, macrophages (nucleus, blue; membrane, green) with fluorescently-labeled nanosponges (red) attached were seeded to the confocal dishes and were visualized using CLSM (Olympus, FV10-ASW).

Macrophages (dispersed in 1 mL RPMI1640 medium) were placed into the confocal dishes and cultured for 4 h under normal incubation conditions (37 °C, 5% CO_2). Then, 10 μL cationic cellulose (polyquaternium-10, 30 mg/mL) was supplemented and shaken for 10 s to make the surface of macrophages positively charged. Subsequently, the macrophages were washed three times with PBS buffer to remove excess polyquaternium-10. And then 0.65 mg of Fe^{III}-TA gated nanosponges were added to the 1 mL treated macrophages and mixed for 10 s. The resulting suspension was washed three times with PBS buffer, and then redispersed in FBS-free RPMI1640 medium for 6 and 12 h, respectively. Finally, the resulting MAGN were fixed (4% paraformaldehyde solution) and stained (DAPI and DiR) for the confirmation of MAGN morphology by confocal microscopy.

Section S12. Live/Dead cell staining

MAGN were seeded in a 24-well plate and incubated for 4 h. Then, the cells were co-

incubated with both Calcein-AM (2×10^{-6} M) and PI (4×10^{-6} M) for 30 min in a humidified atmosphere containing 5% CO_2 at 37 °C. Finally, the cells were washed three times with PBS buffer and observed using CLSM.

Section S13. MAGN viability assay

MAGN and Mφs (served as controls) were seeded in 96-well plates at a density of 5×10^5 cells/well with 100 μL medium (FBS-free RPMI1640) and cultured in a humidified atmosphere containing 5% CO_2 at 37 °C for regular time points. After 0.5, 1, 2, 4, 8, and 12 h, the remaining medium was discarded and another 100 μL fresh medium with 10% cell counting Kit-8 was added to the medium, and the cells were incubated for 30 min. The A450 intensity was measured using an Infinite Nano microplate reader (Tecan Group, Switzerland).

Section S14. Cell migration/invasion assay

Cell migration/invasion assay was used to examine whether MAGM remained tumor-tropic. Unloaded macrophages (Mφs) served as controls. A transwell polycarbonate membrane cell culture insert set (8.0 μm pore sized) was fitted into a 24-well cell culture plate for this study. Invasion assay required coating the upper surface of the inserts with a layer of matrigel beforehand. For experimental groups, B16F10 cells (0.2×10^6 cells) as the lure were seeded to the bottom of each well and cultured overnight. Then, 0.4×10^6 MAGN and normal Mφ (dispersed in 1.0 mL FBS-free RPMI1640 medium) were seeded onto the upper chamber of each insert, respectively. The transmigration process took 24 hours to accomplish under normal incubation conditions (37 °C, 5% CO_2). After that, the transwell inserts were collected, and washed twice with PBS buffer, then fixed with 4% paraformaldehyde for 5 min, followed with twice washing using PBS buffer again and subjected to giemsa staining for 10 min. Those cells that failed to transmigrate (i.e., remained on top of the film) were scraped off with cotton swabs. Optical images of the transmigrated macrophages were captured. Due to giemsa staining, migrated/invaded cells were blue in bright-field images. For each sample, images of different areas were acquired for cell counting to obtain a statistically significant result. The experiment was repeated twice.

Section S15. Interaction of drug released from MAGN with B16F10 cells

DOX internalization was confirmed using flow cytometry and confocal microscopy. For flow cytometry analysis, 1×10^6 B16F10 cells were plated in a 24-well plate and allowed to adhere overnight. Plates were then aspirated, and 1 mL of fresh medium was added to each well. A transwell polycarbonate membrane cell culture insert set (8.0 μm pore sized) was fitted into a 24-well cell culture plate for this study. 1×10^6 MAGN

cells (dispersed in 0.2 mL FBS-free RPMI1640 medium, pH 7.4 and pH 6.0, respectively) were seeded onto the upper chamber of each insert and allowed to co-incubate for 2 h at 37 °C in an incubator. After the stipulated time points, the inserts were removed, medium in the wells was completely aspirated and washed three times with PBS buffer and these cells were detached from plate using 0.25% Trypsin-EDTA solution. After being washed with PBS buffer, these B16F10 cells were analyzed by flow cytometry to test the DOX fluorophore.

For confocal microscopy, 2×10^6 B16F10 cells were plated in a 24-well plate and allowed to adhere for 10 h, transwell inserts were fitted into the plate. 1×10^6 MAGN (dispersed in 0.2 mL FBS-free RPMI1640 medium, pH 7.4 and pH 6.0, respectively) were seeded onto the upper chamber of each insert and allowed to co-incubate for 2 h at 37 °C in an incubator. After the stipulated time points, cells of lower compartment were washed three times with PBS buffer before fixing with 4% paraformaldehyde. The fixed B16F10 cells were stained using DAPI (Ex/EM 340/488 nm) for nucleus and were analyzed using confocal microscopy.

Section S16. Toxicity of MAGN to B16F10 melanoma cells

This assay was used to compare whether MAGN enhanced tumor killing. A transwell polycarbonate membrane cell culture insert set (8.0 μm pore sized) was fitted into a 24-well cell culture plate for this study. B16F10 cells (0.2×10^6 cells) as the lures were seeded to the lower compartment of each well and cultured overnight. Unloaded Mφs served as controls. For the control group, 1×10^6 Mφs with FBS-free RPMI1640 medium (pH 7.4) were added into the upper compartment of each well. For experimental groups, 1×10^6 MAGN (dispersed in 0.2 mL FBS-free RPMI1640 medium, pH 7.4) were seeded onto the upper chamber of each insert. The 24-well cell culture plate was cultured under normal incubation conditions (37 °C, 5% CO_2). After 24h, the bottom B16F10 cells were washed twice with PBS buffer, tested with 10% cell counting Kit-8 (CCK-8) for viability, and the cells were incubated for 2 – 4 h until the color of the control group turned bright yellow. The A450 intensity was measured using a Thermo Scientific V arioskan Flash multimode reader.

In order to compare the tumor-killing ability of the cell patches under different pH conditions, we changed the pH value of the experimental groups. The pH values of culture medium were adjusted to 6.0 (tumor microenvironment) with dilute hydrochloric acid. After that, the experimental groups were co-cultured for 24h and performed CCK-8 tests (as above).

Section S17. Cell viability assay

Mφs were seeded in 96-well plates at a density of 5×10^4 cells/well with 100 μL medium and cultured overnight. Cultured cells were treated with polyquaternium-10.

After 24 h, the remaining medium was discarded and another 100 μL fresh medium with 10% cell counting Kit-8 was added to the medium, and the cells were incubated for 2 – 4 h until the color of the control group turned bright yellow. The A450 intensity was measured using a Thermo Scientific Varioskan Flash multimode reader.

Section S18. *Ex vivo* biodistribution assay

B16F10 tumor-bearing C57Bl/6J mice (n = 3) were utilized to investigate the different biodistribution of DOX between free DOX group and MAGN group. When the tumor volume reached approximately 500 mm^3, free DOX and MAGN were administrated via the tail vein at a dose equivalent to 3 mg/kg of DOX. The mice were killed after 24 h post-injection and the major organs (heart, liver, spleen, lung, and kidney) and tumors were harvested and imaged using an IVIS spectrum small-animal imaging system (IVIS Lumina Series III, PerkinElmer).

Section S19. *In vivo* therapy study

C57Bl/6J mice (aged five weeks) were purchased from Dashuo Laboratory Animal Technology, Ltd. (Chengdu, China) and were kept on a 12 h day/night cycle with free access to food and water. All the experiments and procedures were performed in accordance with the National Institutes of Health guide for the care and use of Laboratory Animals (NIH Publications No. 8023, revised 1978). All animal experiments were approved by the Experimental Animal Management and Ethics Committee of West China Second University Hospital (Approval number: KS2022786). B16F10 cells (5×10^6 cells/mouse) were injected into the right-back of mice in sterile PBS (100 μL). When the tumor volume was approximately 100 ~ 150 mm^3, 20 mice were randomly divided into 4 groups and were i.v. injected with PBS, free DOX, Mφ cells, and MAGN on Day 0 and Day 3, respectively. DOX and MAGN were injected at 3 mg DOX kg^{-1}, and 4×10^6 cells were injected into the mice in Mφ and MAGN groups. The tumor volume was calculated by the following equation: tumor volume = $0.5 \times \text{length} \times (\text{width})^2$, where length ≥ width. The tumor size and body weight of each mouse were measured at every third day for 15 days. Mice were euthanized once the tumor volume was above 1700 mm^3. After 15 days, mice were sacrificed. The tumors and major organs were collected. The formalin-embedded tissues were stained by H&E to identify the pathological change of major organs.

Section S20. Supplementary figures S1–S21

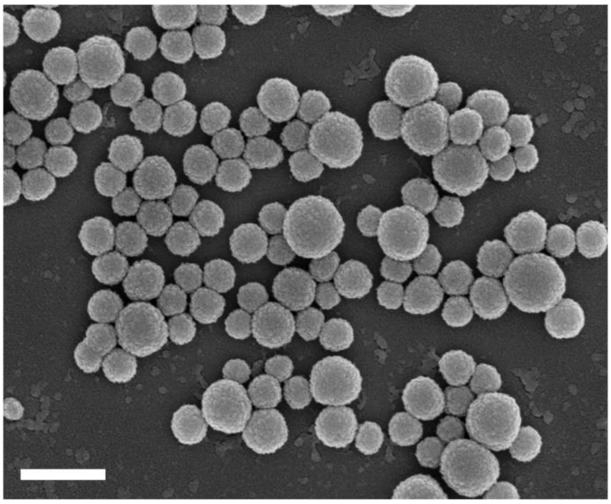

Figure S1. SEM images showing the morphological features of the nanosponges. Scale bar, 1 μm.

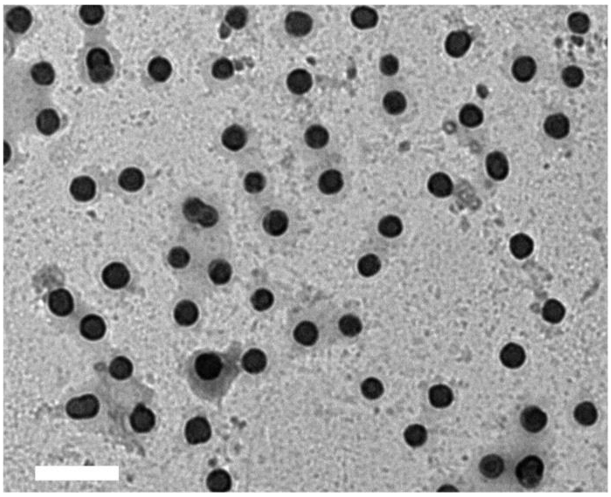

Figure S2. TEM images showing the morphological features of nanosponges. Scale bar, 1 μm.

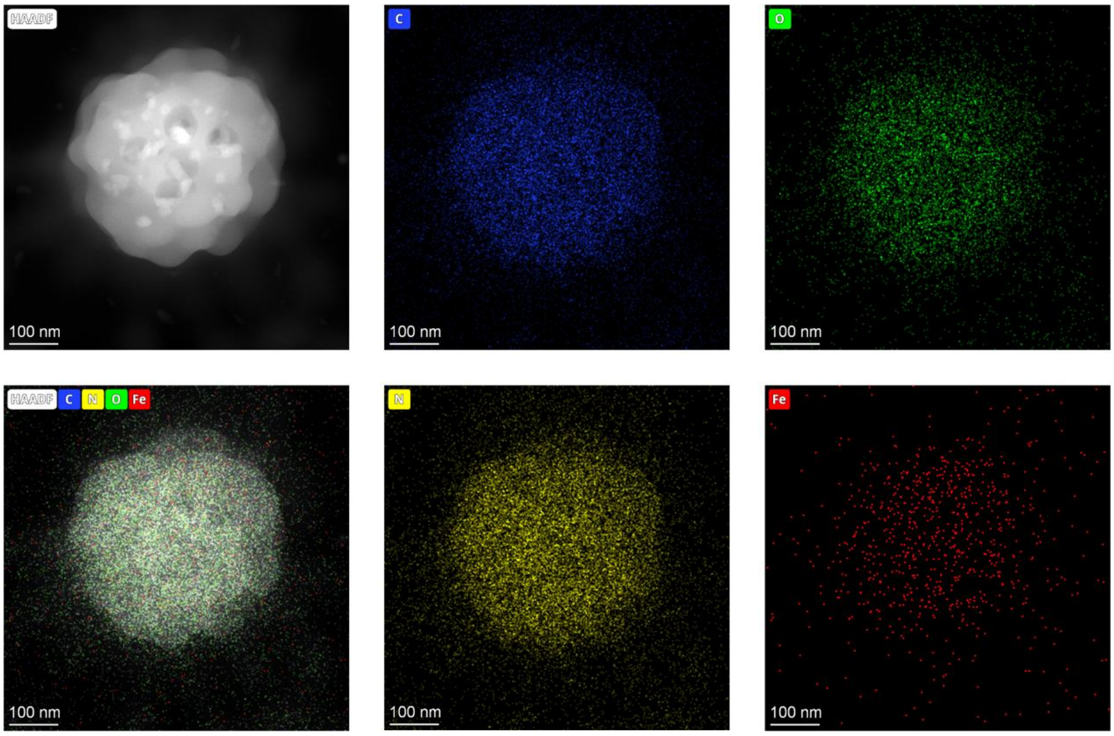

Figure S3. HAADF-STEM image and mapping of the Fe^{III}-TA gated nanosponges. The signals of C, O, N, and Fe were marked in blue, green, yellow, and red, respectively. Scale bars, 100 nm.

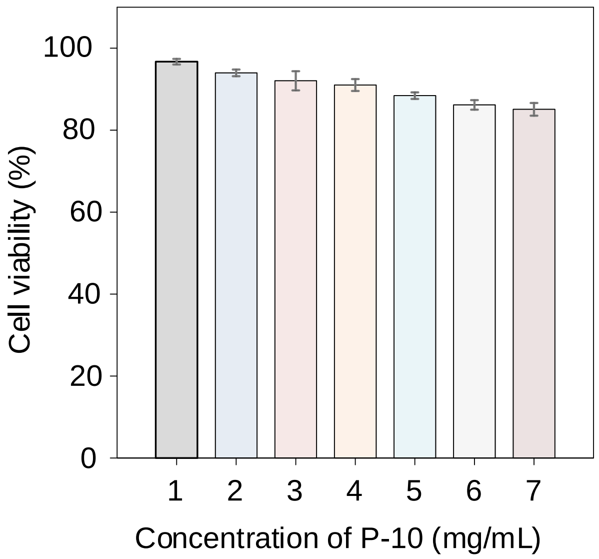

Figure S4. The cell viability of macrophages detected by CCK-8 assay after being incubated with polyquaternium-10 (P-10, 1–7 mg/mL) for 24 h. Data are presented as mean ± SD. (n = 3).

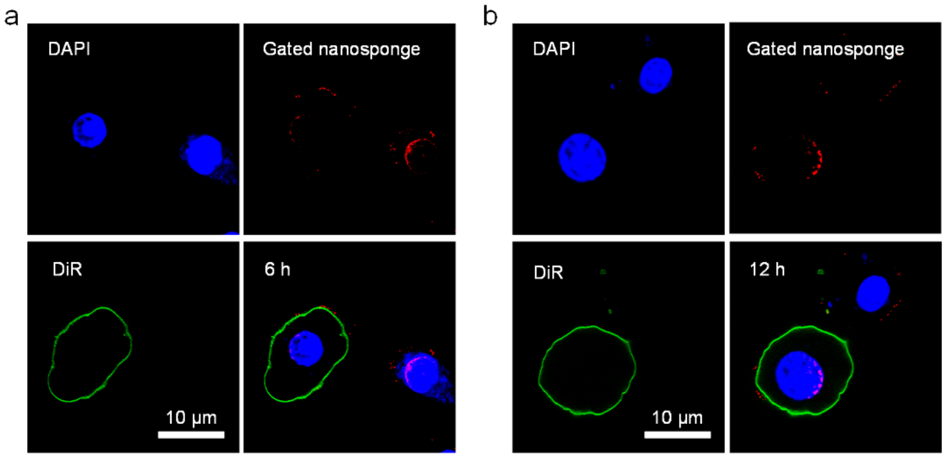

Figure S5. a, b) Confocal micrographs of Mφs (cell nucleus, blue; membrane, green) displaying nanosponges with DOX (red) after 6 h and 12 h, respectively. Scale bars, 10 μm.

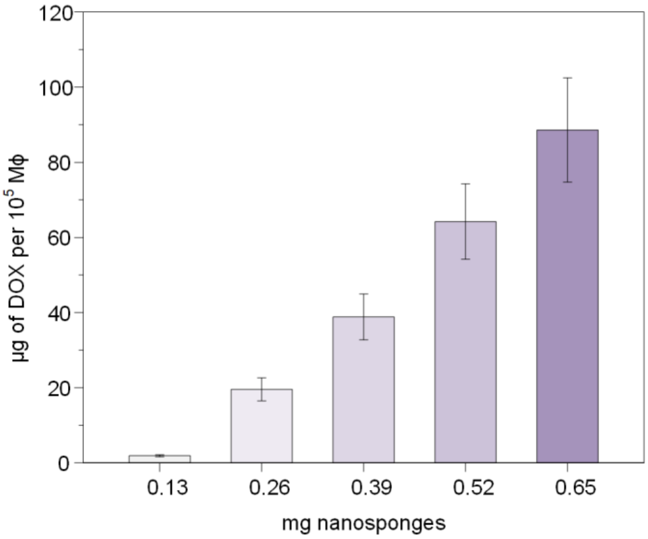

Figure S6. Drug loading capacity of the nanosponges at different nanosponge–to–macrophage ratios.

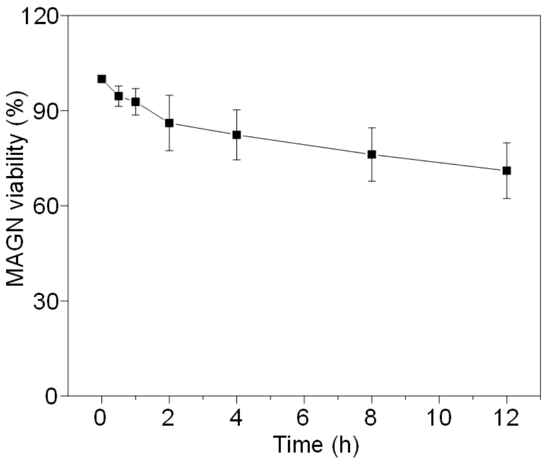

Figure S7. Cell viability test of the MAGN after different incubation time.

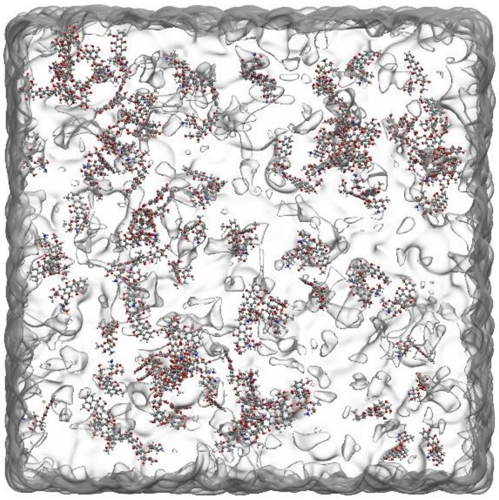

Figure S8. The initial state of the simulation boxes packed with 20 TA molecules and 80 DOX molecules at pH 7.4.

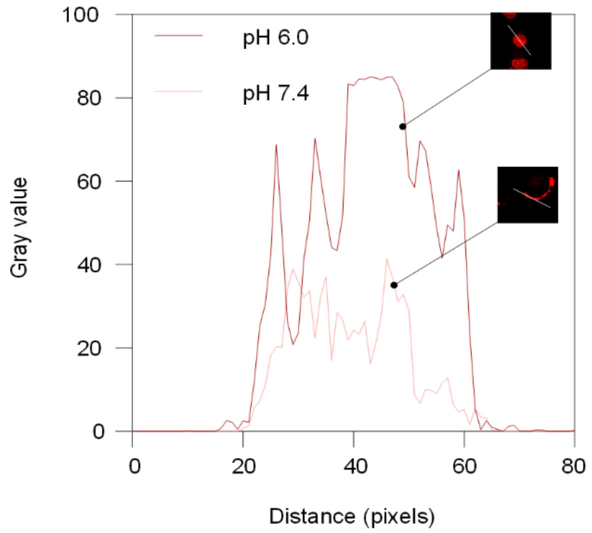

Figure S9. Analysis of DOX fluorescence intensity of B16F10 cells under different pH conditions in Figure 4e. Inset: confocal photographs of B16F10 cells with DOX.

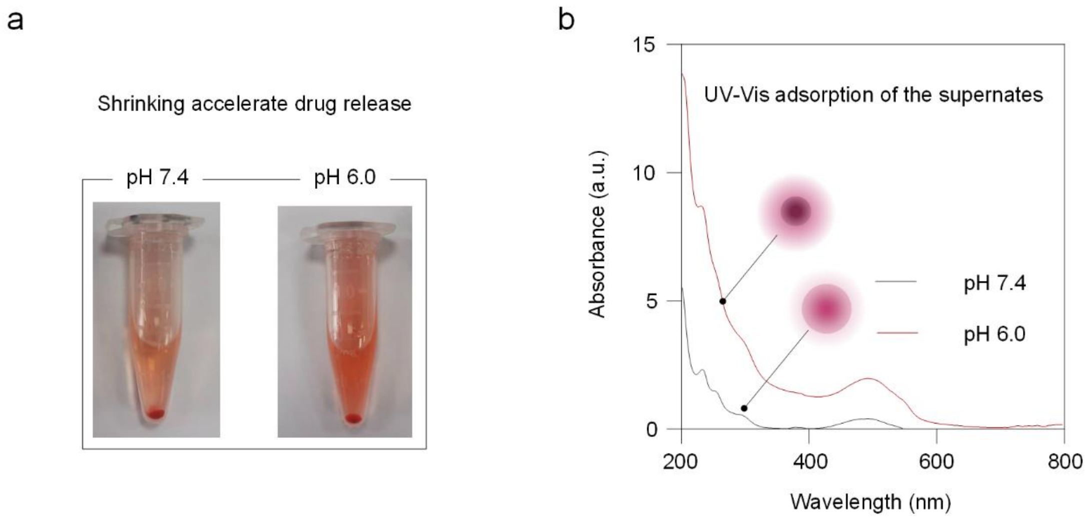

Figure S10. a) Photographs of centrifuged samples of ungated nanosponges after shaking in pH 7.4 and pH 6.0 PBS in Eppendorf tubes. b) UV absorption spectra of ungated nanosponges as a function of pH.

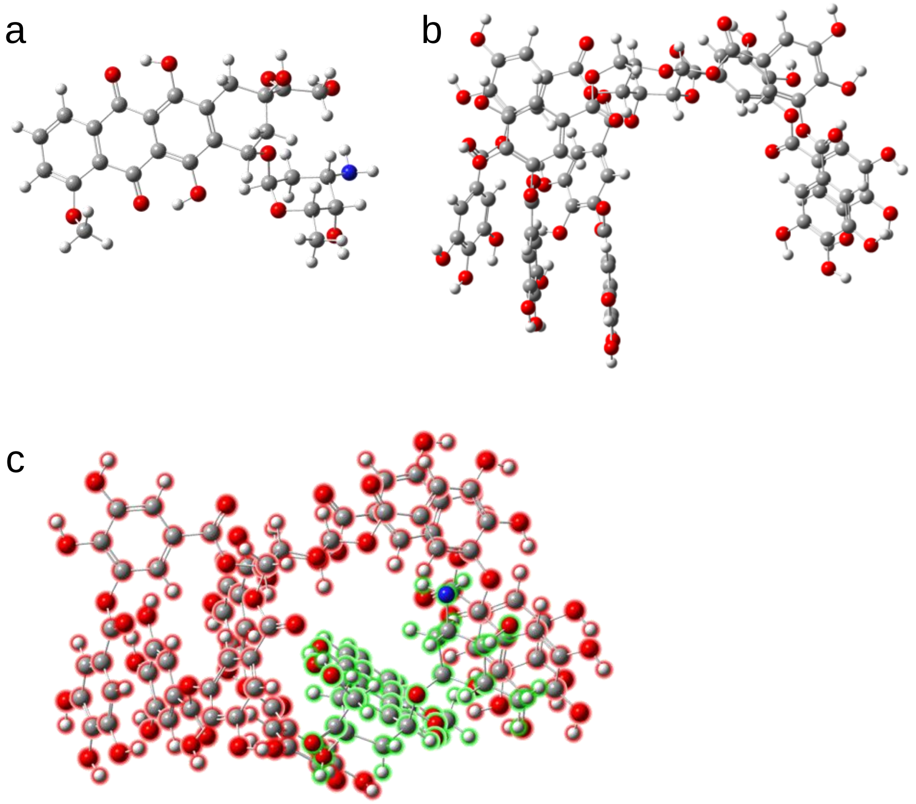

Figure S11. a) Structure diagram of DOX molecules at pH 7.4, blue balls represent N, red represents O, gray represents C and white represents H, respectively. b) Structure diagram of TA molecules at pH 7.4. c) Interaction diagram of TA molecules and DOX molecules at pH 7.4, the green aperture represents DOX and red aperture represents TA.

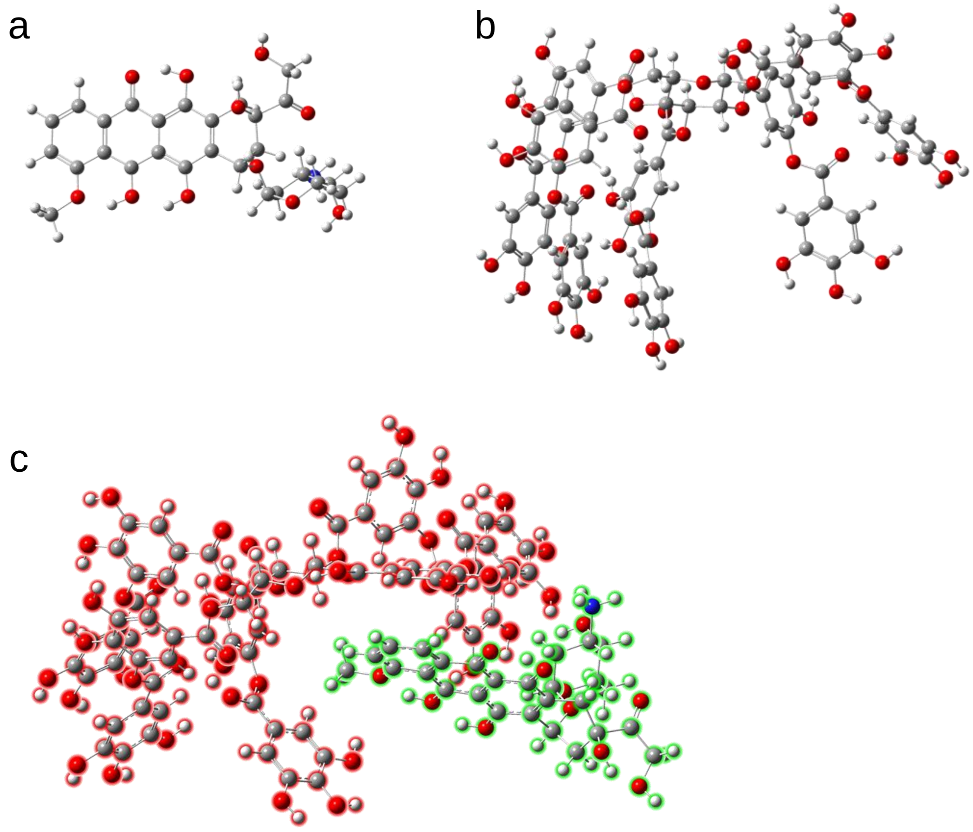

Figure S12. a) Structure diagram of protonated DOX molecules at pH 6.0, blue balls represent N, red represents O, gray represents C and white represents H, respectively. b) Structure diagram of protonated TA molecules at pH 6.0. c) Interaction diagram of protonated TA molecules and protonated DOX molecules at pH 6.0, the green aperture represents protonated DOX and red aperture represents protonated TA.

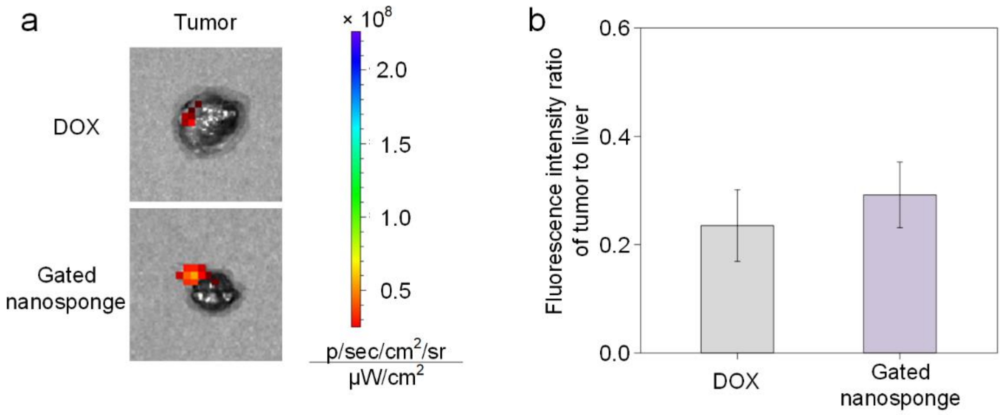

Figure S13. a) In vivo imaging system (IVIS) images of tumors 24 h after i.v. injection of free DOX and the gated nanosponges groups, respectively. b) Fluorescence intensity ratios of the tumor to the liver for the free drug and gated nanosponge groups.

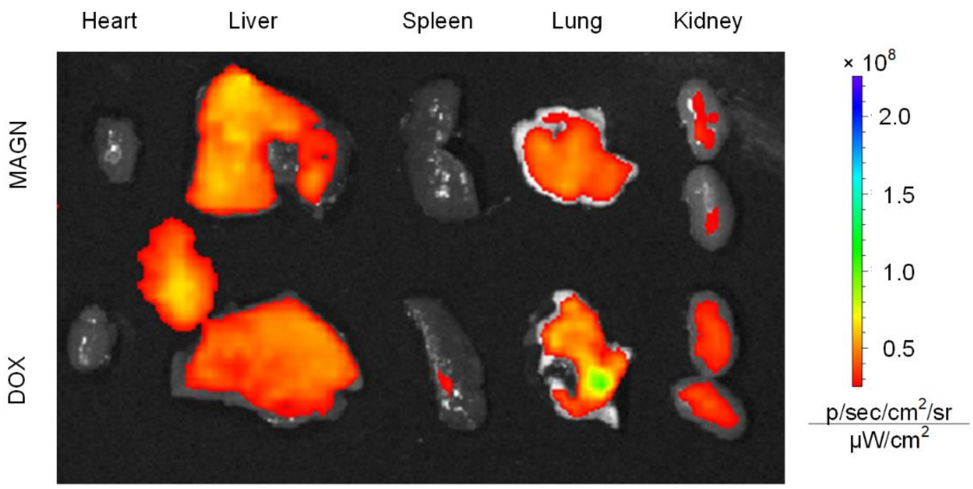

Figure S14. IVIS images of heart, liver, spleen, lung, and kidney after injection of MAGN and DOX for 24 h.

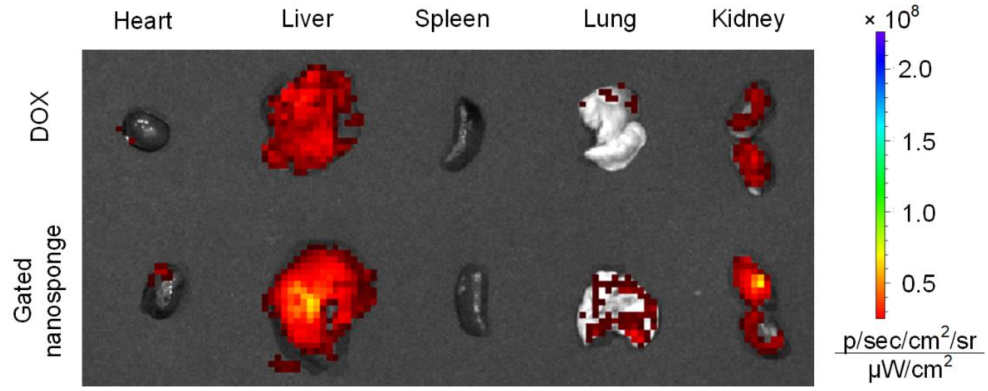

Figure S15. IVIS images of heart, liver, spleen, lung, and kidney after injection of the free DOX and the gated nanosponges groups for 24 h.

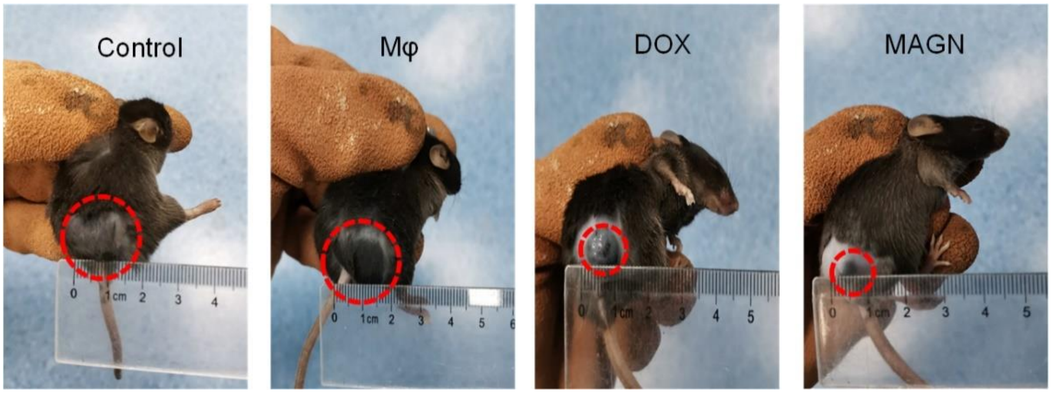

Figure S16. Photographic images of mice bearing B16F10 tumors with treatments of control, Mφ, DOX and MAGN.

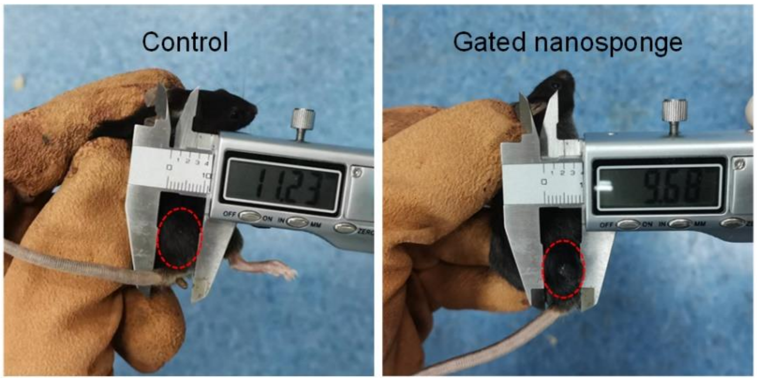

Figure S17. Photographic images of mice bearing B16F10 tumors with treatments of control and the Fe^{III}-TA networks gated nanosponges groups.

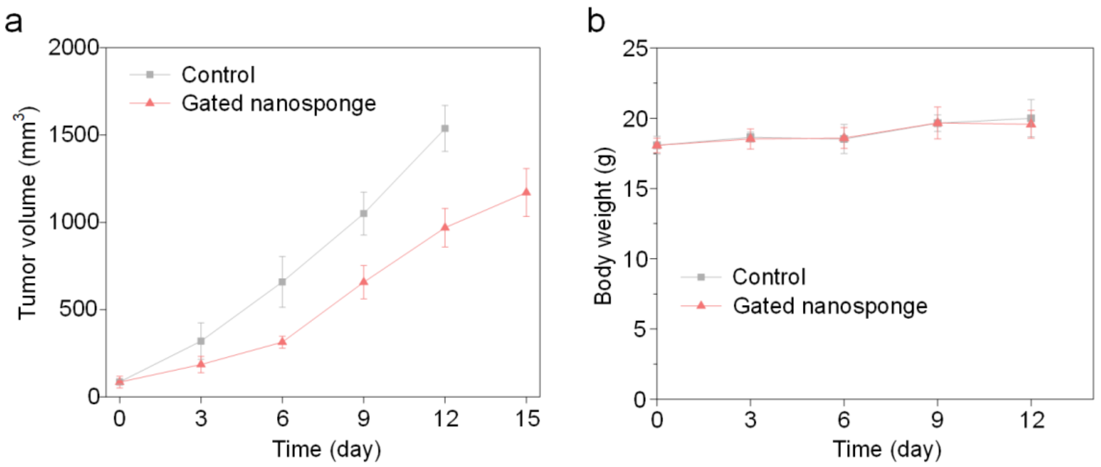

Figure S18. a) Tumor growth profiles after treatment with control and the Fe^{III}-TA networks gated nanosponges groups. b) Plot of body weight versus time in tumor-bearing mice.

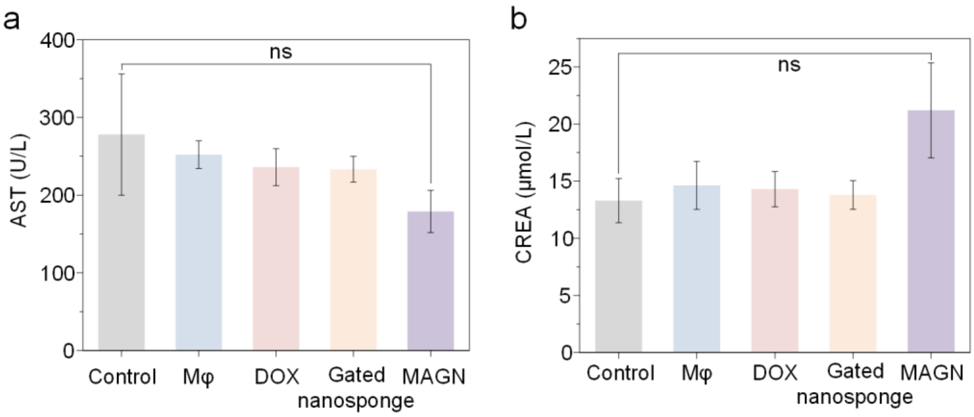

Figure S19. Mice were euthanized on Day 15 for blood biochemistry analysis: a) AST levels, and b) CREA levels. For MAGN group, all the indices were in the normal ranges (ns represents no significant difference).

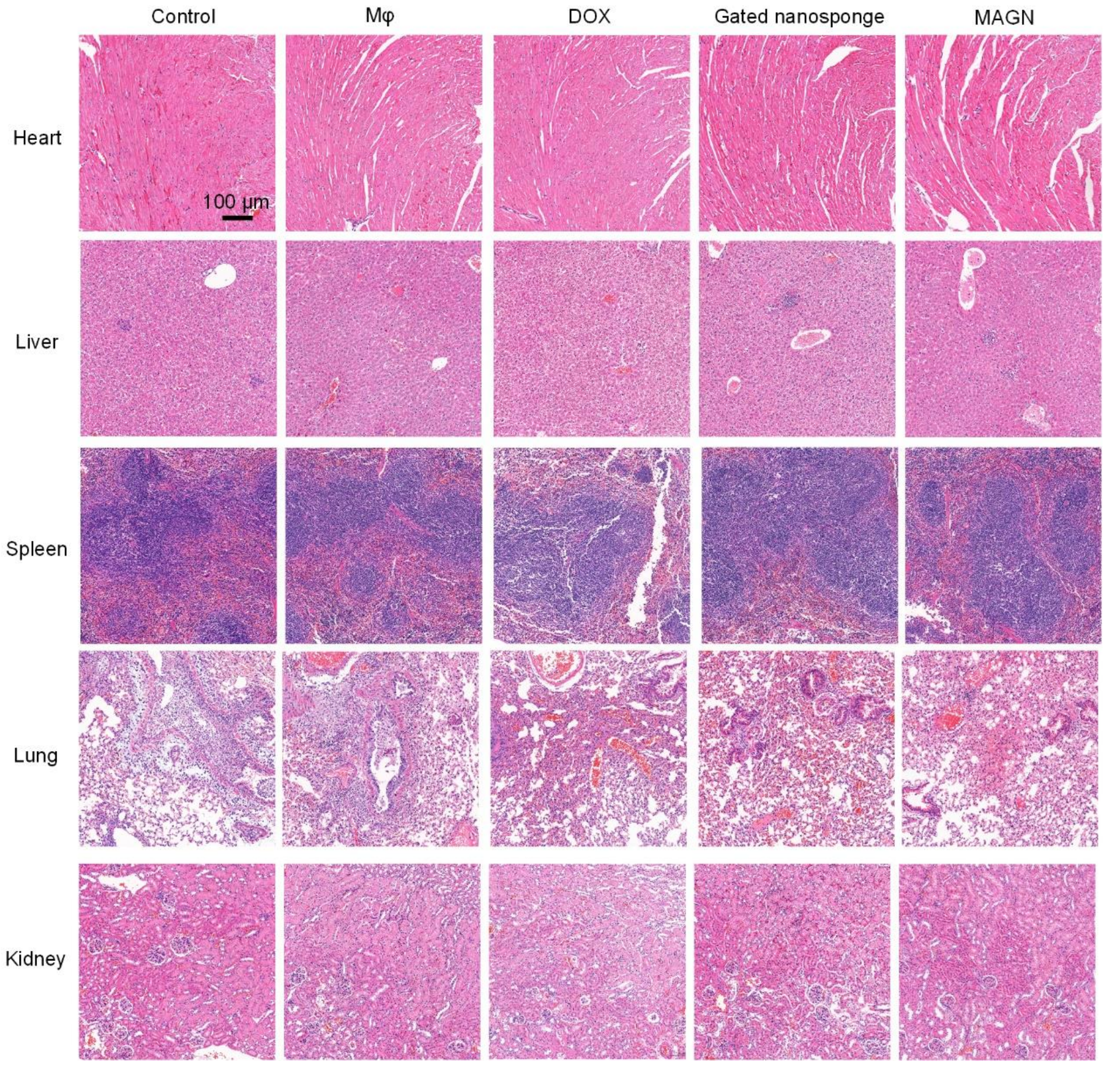

Figure S20. Representative images of H&E staining of major organs collected on Day 15 post-treatments, no pathological changes were observed for the MAGN group. Scale bars, 100 μm.

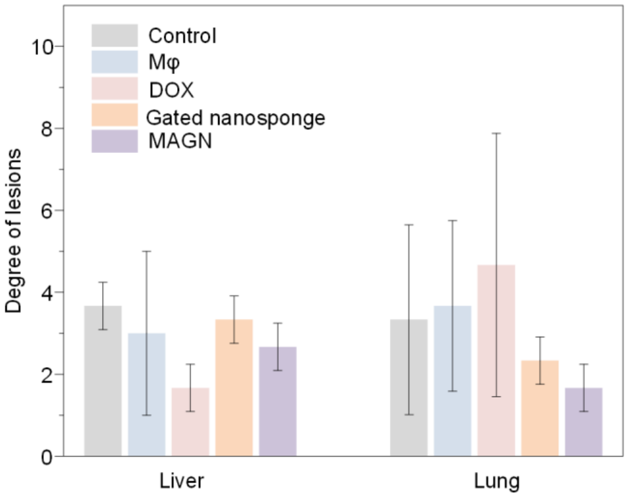

Figure S21. Lesions degree evaluation of the liver and lung after different treatments. The standard for evaluation: 0, no lesion; 1: minor lesion; 2: mild lesion; 3: moderate lesion; 4: severe lesion.

Section S21. Supplementary tables S1–S2

Table S1. The polydispersity index (PDI) of the nanosponges evaluated by DLS at different pH values.

Nanosponge	Polydispersity index (PDI)
pH 7.4	0.34
pH 6.5	0.46
pH 5.5	0.33
pH 4.5	0.23
pH 3.2	0.32

Table S2. Mice were euthanized on Day 15 for blood routine examination.

	Control	DOX	Gated nanosponge	Mφ	MAGN
WBC (× 10^9/L)	1.15 ± 0.78	2.23 ± 0.9	2.5 ± 0.26	1.05 ± 0.07	2.47 ± 1.09
Lymph# (× 10^9/L)	0.05 ± 0.07	1.13 ± 1.04	0.83 ± 1.01	0.5 ± 0.57	0.7 ± 0.87
Mon# (× 10^9/L)	0.15 ± 0.07	0.17 ± 0.12	0.23 ± 0.12	0.05 ± 0.07	0.23 ± 0.15
Gran# (× 10^9/L)	0.95 ± 0.64	0.93 ± 1.18	1.43 ± 1.0017	0.5 ± 0.57	1.53 ± 1.36
RBC (× 10^{12}/L)	8.78 ± 0.7	8.07 ± 0.088	8.45 ± 0.62	8.99 ± 0.76	8.38 ± 0.50
HGB (g/L)	163 ± 14.14	147 ± 2	156.67 ± 16.86	165 ± 14.14	153 ± 17.34
HCT (%)	34.75 ± 2.76	32.33 ± 0.29	33.83 ± 2.74	36.15 ± 2.62	33.5 ± 2.72
MCV (fL)	39.65 ± 0.07	40.13 ± 0.40	40.1 ± 0.53	40.25 ± 0.49	40 ± 0.96
MCH (pg)	18.5 ± 0.14	18.17 ± 0.15	18.47 ± 0.64	18.3 ± 0	18.17 ± 0.97
MCHC (g/L)	468.5 ± 3.53	454.33 ± 3.21	462 ± 15.52	455.5 ± 6.36	455.33 ± 16.62
RDW (%)	10.8 ± 0.57	11.5 ± 0.72	10.73 ± 0.29	11.25 ± 0.49	10.77 ± 0.51
PLT (× 10^9/L)	979.5 ± 136.47	632.67 ± 256.41	621.33 ± 370.03	288 ± 135.76	643.33 ± 344.57
MPV (fL)	5 ± 0.14	5.47 ± 0.46	5.33 ± 0.25	5.6 ± 0	5.23 ± 0.25
PDW	16.1 ± 0	16.067 ± 0.21	16.3 ± 0.35	16.4 ± 0	16.2 ± 0.26
PCT (%)	0.49 ± 0.08	0.34 ± 0.12	0.33 ± 0.19	0.16 ± 0.076	0.33 ± 0.17

Section S22. Supplementary references

1. J. Pan, G. Gong, Q. Wang, J. Shang, Y. He, C. Catania, D. Birnbaum, Y. Li, Z. Jia, Y. Zhang, N. S Joshi, J. Guo, *Nat. Commun.* **2022**, *13*, 2117.
